# Multimodal deep learning enhances genomic risk prediction for cardiometabolic diseases

**DOI:** 10.1093/bib/bbag550

**Published:** 2026-10-08

**Authors:** Taiyu Zhu, Upamanyu Ghose, Héctor Climente-González, Joanna Howson, Sile Hu, Alejo Nevado-Holgado

**Affiliations:** Department of Psychiatry, University of Oxford, Warneford Lane, Warneford Hospital, OX3 7JX Oxford, United Kingdom; Department of Psychiatry, University of Oxford, Warneford Lane, Warneford Hospital, OX3 7JX Oxford, United Kingdom; Novo Nordisk Research Centre Oxford, Innovation Building, Roosevelt Drive, Old Road Campus, OX3 7FZ Oxford, United Kingdom; Novo Nordisk Research Centre Oxford, Innovation Building, Roosevelt Drive, Old Road Campus, OX3 7FZ Oxford, United Kingdom; Novo Nordisk Research Centre Oxford, Innovation Building, Roosevelt Drive, Old Road Campus, OX3 7FZ Oxford, United Kingdom; Department of Psychiatry, University of Oxford, Warneford Lane, Warneford Hospital, OX3 7JX Oxford, United Kingdom

**Keywords:** cardiometabolic diseases, deep learning, genetic risk prediction, genome-wide variants

## Abstract

Cardiometabolic diseases (CMDs) are multifactorial disorders influenced by numerous genetic variants and their complex interactions. Although recent studies have advanced the understanding of genetic risk prediction, current approaches predominantly rely on linear models that may not fully capture the complex, nonlinear relationships between genetic factors. Here, we present DeepGP (Deep learning-based Genome-wide Predictor), a novel multimodal deep learning framework that incorporates bidirectional state space models to predict CMD risk using genome-wide variants and demographic data. We conducted extensive experiments to evaluate DeepGP’s performance. First, in simulation studies incorporating joint genetic and environmental interactions, we demonstrated DeepGP’s superior prediction performance across varying levels of heritability. When evaluated on eight CMDs in cohorts of European ancestry from the UK Biobank (UKB), DeepGP achieved significantly higher accuracy compared with conventional polygenic risk scores and machine learning methods. Model interpretability analysis identified both well-established genes and potential new signals contributing to the risk of the disease. Evaluation within UKB subpopulations of African and Caribbean ancestry provided preliminary evidence of within-resource transferability for type 2 diabetes. These results demonstrate the potential of deep learning technologies to enhance genetic risk stratification for complex diseases, while underscoring the need for external validation in more diverse cohorts.

## Introduction

Cardiometabolic diseases (CMDs) are major contributors to global morbidity and mortality, underscoring the need for effective strategies for early risk stratification and prevention [1]. Many CMDs are polygenic, with risk distributed across numerous genetic variants throughout the genome. Genome-wide association studies (GWAS) [2] and large-scale resources such as the UK Biobank (UKB) [3–5] have enabled the identification of disease-associated variants and supported the development of genomic risk prediction approaches.

A major application of GWAS is the polygenic risk score (PRS), which aggregates the effects of many variants into a single estimate of genetic susceptibility [6]. PRS has been applied to multiple CMDs in large biobank datasets [7–15]. However, conventional PRS frameworks are largely based on linear or logistic regression (LR) models with additive assumptions. These approaches may not fully capture nonadditive and nonlinear effects, including gene–gene and gene–environment interactions, and often show reduced portability across populations with different linkage disequilibrium (LD) structures and genetic architectures [16, 17].

Machine learning methods have therefore attracted interest as alternatives for genomic prediction, particularly for modelling small-effect variants and more complex genotype–phenotype relationships [18–20]. Among these, LightGBM (LGBM) has shown strong performance on high-dimensional genomic data [21–24]. Deep learning has also shown promise in genomics, including variant effect prediction and disease classification [25–33]. However, applying deep learning at genome-wide scale remains challenging because genetic data contain millions of variants, creating substantial computational and memory constraints. This challenge is particularly relevant for CMD risk prediction, where relatively few studies have evaluated genome-scale deep learning approaches or their transferability across ancestries.

To address these limitations, we developed DeepGP (Deep learning-based Genome-wide Predictor), a multimodal deep learning framework for CMD risk prediction from genome-wide variants and demographic covariates. DeepGP uses bidirectional (Bi-SSM) modules based on Mamba [34] to model long-range dependencies while maintaining linear computational scaling, making genome-wide learning more tractable than with conventional Transformer-based approaches. The framework processes SNPs chromosome by chromosome and integrates them with covariate information to generate disease risk predictions. We evaluated DeepGP in simulation studies, across eight CMDs in UKB European-ancestry cohorts, and in type 2 diabetes prediction in African and Caribbean ancestry cohorts to assess predictive performance, interpretability, and transferability.

## Results

### Overview of methods and workflow

Figure 1 summarizes the data processing pipeline and the proposed deep learning framework. Participants were first categorized into European ($n=364{\,}541$), African, and Caribbean cohorts based on self-reported ancestry and projection onto the principal components (PCs) (Supplementary Fig. 1). SNP quality control (QC) was then performed using an existing pipeline [35], followed by removal of related participants up to the third degree to reduce confounding from cryptic relatedness. SNP data were extracted from UKB imputed genotypes [3].

**Figure 1:**
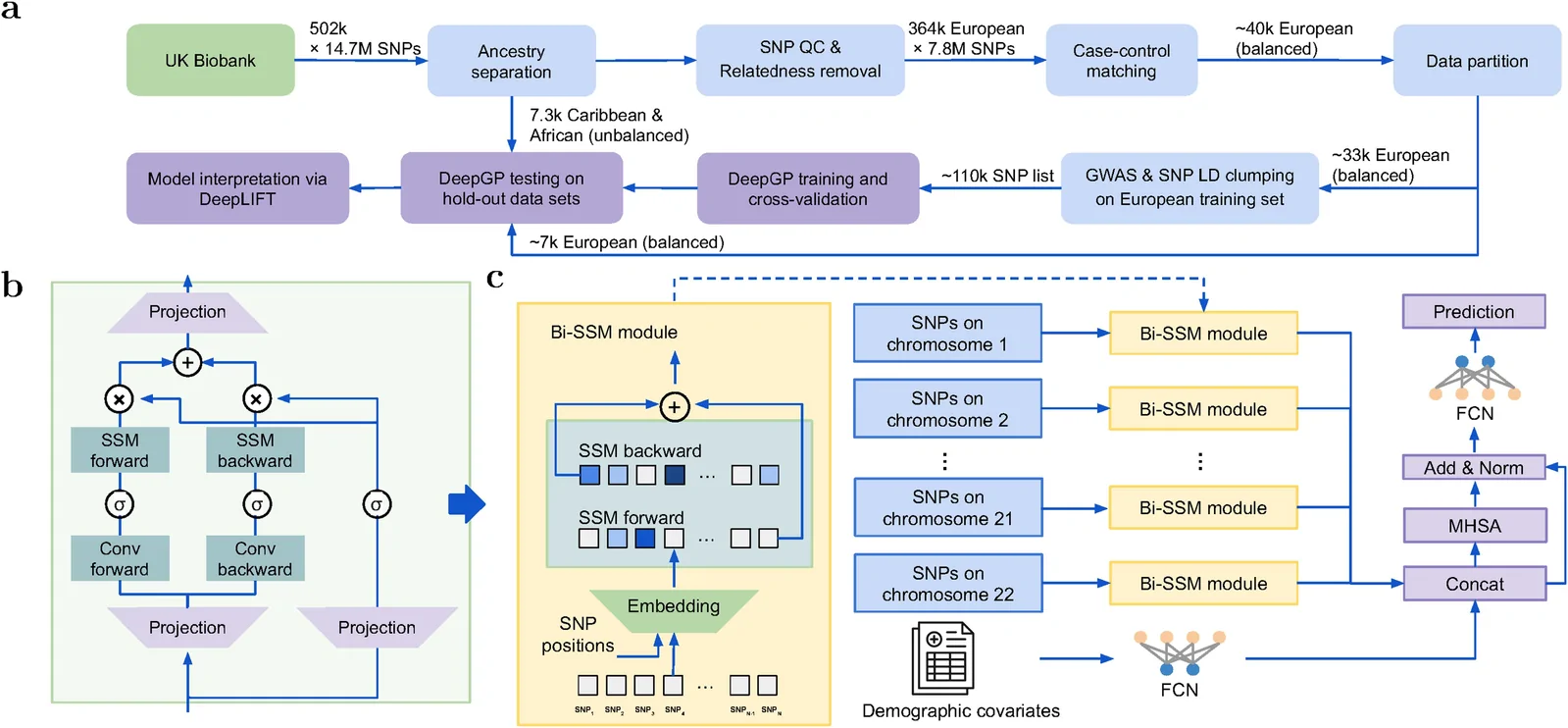
Overview of the proposed disease risk prediction model. (a) Data preprocessing pipeline to extract genotype profiles from the UKB. The input data includes imputed genotype data, and covariates reflecting environmental effect, including sex, age, and six principal components (PCs) of population structure. (b) Architecture of a Bi-SSM, which consists of bidirectional selection mechanism to capture the complex interactions and long-range dependencies between SNPs within each chromosome. *σ* is a nonlinear activation function. (c) Architecture of DeepGP for predicting the risk of CMDs. The SNP data from each of the 22 autosomal chromosomes are processed by separate Bi-SSM modules, while the covariates are processed by an FCN branch. The outputs from these parallel processing paths are then integrated through a lightweight MHSA module and a final FCN layer to generate risk predictions.

We next performed case-control matching to create balanced European training and testing sets, while retaining the African and Caribbean cohorts for within-resource assessment of generalizability and transferability. GWAS and LD clumping were then conducted in the European training set to select an independent leading SNP from each LD block, reducing redundancy from correlated variants and lowering input dimensionality to fit computational constraints.

The processed SNP data were combined with demographic covariates, including sex, age, and six ancestry PCs from UKB. DeepGP was then trained, cross-validated, and evaluated on the hold-out testing set and two validation cohorts. Model interpretability was assessed using DeepLIFT [36], which assigns feature importance scores through backpropagation of activation.

To learn hidden structure from SNP data, DeepGP uses Bi-SSM modules to extract features in both forward and backward directions (Fig. 1b), inspired by bidirectional recurrent neural networks for sequence analysis [37]. Separate Bi-SSM modules are applied to each of the 22 autosomal chromosomes (Fig. 1c), while covariates are processed by a fully connected network (FCN). This chromosome-wise design constrains most sequence modelling to intra-chromosomal context, where LD and cis-regulatory structure are most directly represented, while allowing cross-chromosome information to be integrated at the embedding level through the output attention layer. The resulting hidden representations are concatenated and passed through output layers, including a multi-head self-attention (MHSA) module, to generate final predictions.

#### Predictive performance in simulation studies

We generated synthetic phenotypes based on imputed genotype data, enabling evaluation under varying genetic architectures and interaction patterns. DeepGP was then compared with baseline methods, including PRS and LGBM.

Specifically, we used LD-clumped SNPs from the type 2 diabetes dataset, retained the same balanced European training and testing split (Supplementary Table 1), and followed the same training, cross-validation, and evaluation procedure shown in Fig. 1a. Phenotypes were simulated as a combination of genetic effects, gene–gene interactions, gene–environment interactions, and random error from unobserved environmental factors. After rescaling, total phenotypic variance was set to 1, with the variance of genetic effects corresponding to SNP heritability ($h^{2}_{\mathrm{snp}}$). The variances of gene–gene and gene–environment interactions were fixed at 0.2, while $h^{2}_{\mathrm{snp}}$ was varied from 0.1 to 0.6. For each $h^{2}_{\mathrm{snp}}$ value, we performed 10 independent simulations with different random seeds.

Figure 2 shows the area under the receiver operating characteristic curve (AUROC) for each method across simulated scenarios. DeepGP, LGBM, and PRS were trained and tested on identical datasets with the same SNP and covariate inputs. Statistical significance of performance differences was assessed using *t*-tests or Mann–Whitney U tests [38], depending on the normality of AUROC differences.

**Figure 2:**
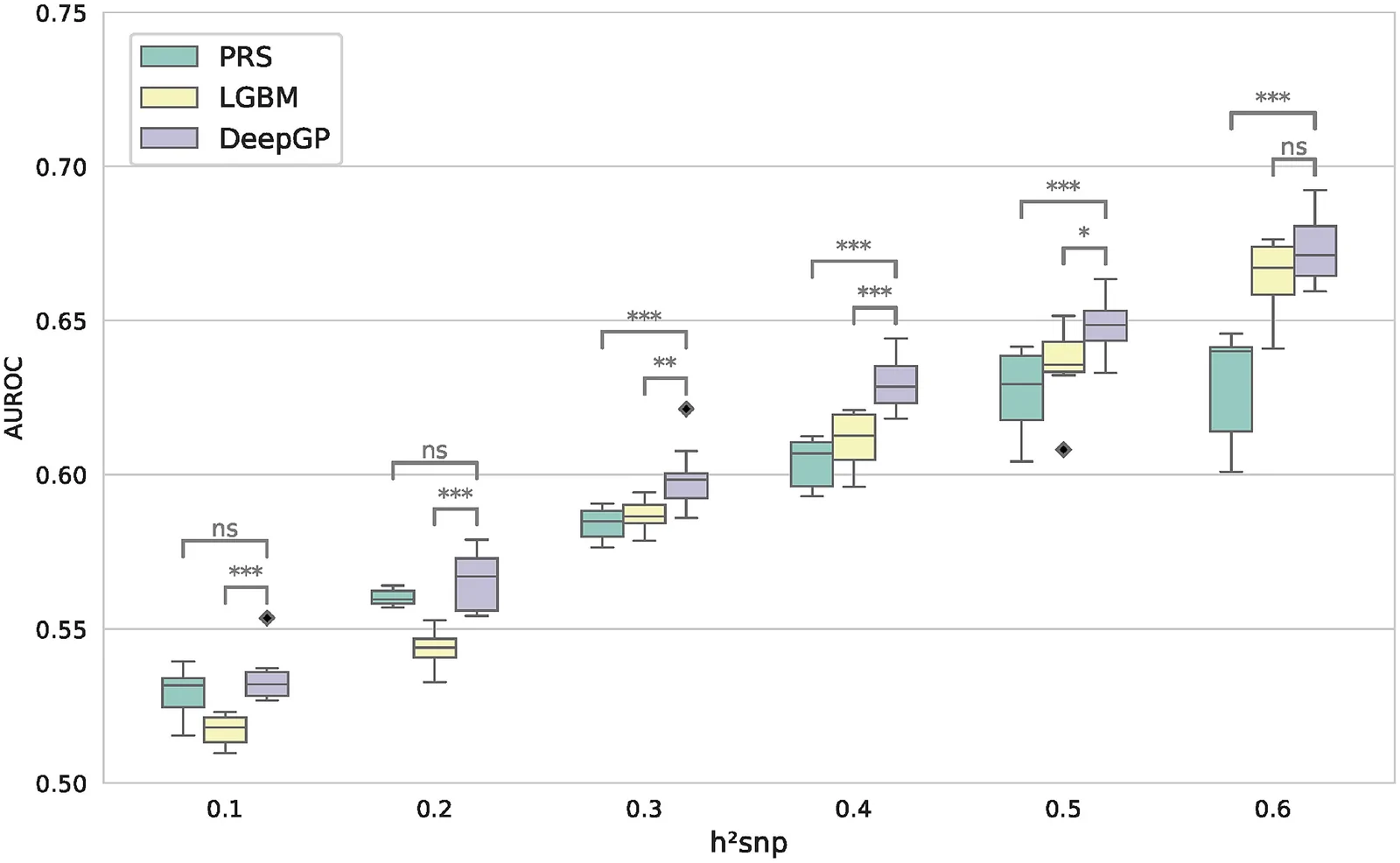
Predictive performance comparison across SNP heritability thresholds. AUROC for DeepGP, LGBM, and standard PRS plotted against varying levels of SNP heritability ($h^{2}_{\mathrm{snp}}$). Diamond markers represent outliers. Statistical significance: $^{***} P<.001$, $^{**} P<.01$, $^{*} P<.05$.

DeepGP consistently outperformed the baseline methods across all SNP heritability settings. The largest gains were observed at moderate heritability ($h^{2}_{\mathrm{snp}}=0.3$–0.5), where DeepGP showed a statistically significant advantage over both LGBM and PRS. As heritability increased to 0.6, the performance gap between DeepGP and LGBM narrowed. Because the simulations included gene–gene and gene–environment interaction terms by design, these results are consistent with, but do not by themselves isolate, the contribution of the Bi-SSM module to modelling nonlinear relationships. To more directly isolate this contribution, we performed an additional controlled simulation in which SNP heritability and the proportion of causal variants were held fixed while the strengths of gene–gene and gene–environment interactions were varied separately, comparing full DeepGP with a unidirectional SSM (Supplementary Fig. 2). Across both mechanisms, full DeepGP consistently outperformed the unidirectional model, and both models improved as interaction variance increased; the advantage of the bidirectional model was approximately constant across interaction strengths. We therefore added a simulation-based comparison between DeepLIFT attribution scores and $\chi ^{2}$ statistics in the Supplementary Information (Supplementary Fig. 3) to illustrate how model attribution can prioritize interaction-involved SNPs that have weaker marginal association.

### Enhanced risk prediction for cardiometabolic diseases

We evaluated DeepGP and baseline methods to predict eight CMDs in the UKB: atherosclerosis, atrial fibrillation, cardiovascular disease, coronary artery disease, heart failure, myocardial infarction, stroke, and type 2 diabetes. For fair evaluation, we used balanced hold-out testing data with equal numbers of cases and controls (Supplementary Table 1). Complementary analyses under natural (imbalanced) prevalence are also provided, with sample sizes reported in Supplementary Table 2 and the PRS-CSx [39] comparison reported in Supplementary Fig. 7 and Table 5.

Table 1 presents the accuracy and AUROC results for DeepGP, LGBM, and PRS, where the best value for each metric is shown in bold. DeepGP consistently achieved the highest accuracy and AUROC scores across all diseases compared with both baseline methods. Additional metrics, including precision, sensitivity, and specificity, are provided in Supplementary Table 3, where DeepGP demonstrated the best performance in all metrics for atherosclerosis and stroke. For the remaining diseases, DeepGP maintained the best trade-off between precision, sensitivity, and specificity, as indicated by its highest AUROC. We further compared performance with a broader range of traditional machine learning and deep learning baseline methods, including CNN, dense neural networks (Dense), multilayer perceptron, random forest, and SNP-level LR models, as shown in Supplementary Fig. 4.

**Table 1:** Accuracy and AUROC of genomic risk prediction on hold-out testing data of European ancestry

Phenotype	Accuracy			AUROC		
	DeepGP	LGBM	PRS	DeepGP	LGBM	PRS
Atherosclerosis	**68.6**	64.7	65.7	**0.75**	0.70	0.70
Atrial fibrillation	**67.6**	65.7	65.9	**0.74**	0.71	0.70
Cardiovascular disease	**67.1**	63.8	64.4	**0.74**	0.69	0.68
Coronary artery disease	**67.4**	63.9	63.5	**0.74**	0.68	0.69
Heart failure	**67.1**	62.8	62.7	**0.72**	0.68	0.66
Myocardial infarction	**69.0**	65.8	62.9	**0.76**	0.71	0.67
Stroke	**63.2**	59.6	57.9	**0.68**	0.62	0.60
Type 2 diabetes	**60.4**	59.5	57.7	**0.64**	0.61	0.60

The second and third most substantial improvements were observed for stroke and heart failure, with a reduction in misclassified cases of 12.6% and 11.8%, respectively, compared with PRS (Supplementary Eq. 1). These three diseases have the smallest number of cases among the eight diseases studied (Supplementary Table 1), suggesting that DeepGP may be robust in some lower-case-count settings. However, because this observation is based on naturally occurring sample-size differences across diseases rather than a controlled down-sampling experiment, it should be interpreted cautiously.

### Model interpretability and genetic insights

To investigate the contribution of each SNP towards the prediction outputs, we performed DeepLIFT, a method for interpreting deep learning models by backpropagating the contributions of all neurons in the network to every feature of the input. This approach allowed us to derive feature importance scores for each input SNP. These scores should be interpreted as model attributions rather than formal association statistics or evidence of causality. We then compared the DeepLIFT feature-importance scores with association statistics from our GWAS conducted on the training subset following a standard pipeline [33].

The scatter plots in Fig. 3 depict the relationships between the DeepLIFT feature importance scores and the Chi-square statistics for each SNP across the eight CMDs. The scatter plots visualize the concordance between the feature importance scores derived from the deep learning model and the statistical significance of the SNPs determined by our GWAS analysis. The vertical blue line represents the Chi-square statistic corresponding to the widely used GWAS *P*-value threshold of $5 \times 10^{-8}$, which is commonly employed to identify significant associations.

**Figure 3:**
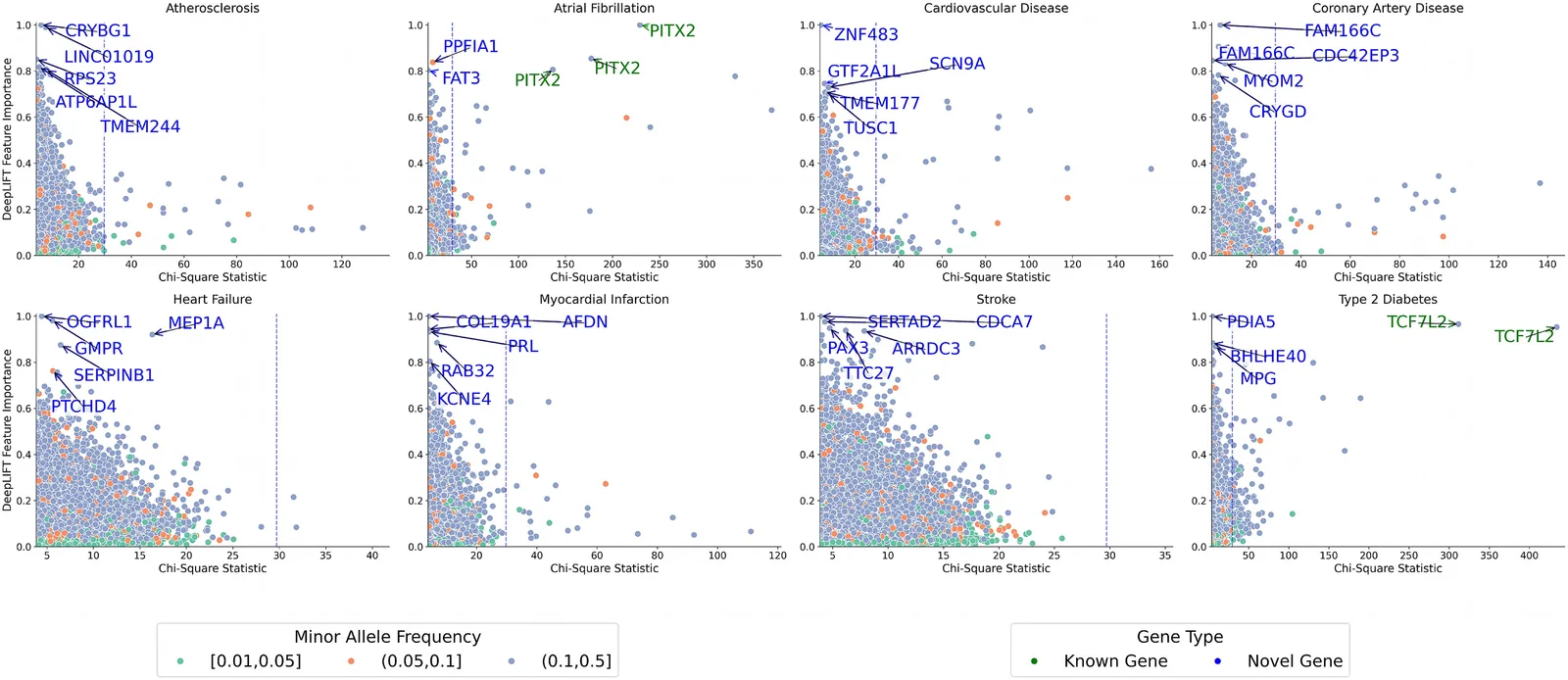
Comparison of feature importance derived from DeepLIFT and $\chi ^{2}$ statistics for the considered CMDs. Points are categorised by minor allele frequency, and the vertical dashed line indicates the $\chi ^{2}$ statistic corresponding to GWAS *P*-value threshold of $5 \times 10^{-8}$. The top five SNPs with the highest feature importance scores are annotated with their nearest genes and labelled on the plots.

In atrial fibrillation and type 2 diabetes, the top GWAS loci, *PITX2* and *TCF7L2*, respectively, also received high DeepLIFT attributions [40, 41], both extensively validated across diverse populations. At loci surpassing the GWAS *P*-value threshold, DeepLIFT attribution magnitudes showed a positive correspondence with GWAS $\chi ^{2}$ statistics. For coronary artery disease, this correspondence was threshold-dependent: the rank concordance (Spearman *ρ*) between DeepLIFT scores and $\chi ^{2}$ statistics rose from $\rho \approx 0.03$ across all nominally associated SNPs to *ρ =0.71* at the genome-wide-significance threshold (Supplementary Fig. 5). However, several loci with high $\chi ^{2}$ statistics had lower DeepLIFT scores, particularly for atherosclerosis, coronary artery disease, and myocardial infarction. We interpret these cases as loci with strong marginal association that may contribute less to the trained model’s multivariable prediction once correlated SNPs, covariates, and nonlinear combinations of features are considered. DeepGP also prioritized SNPs near genes that did not reach significance in the conventional GWAS we conducted. For example, SNPs near *PDIA5* and *MPG* received high attribution for type 2 diabetes. Although *PDIA5* has functions plausibly relevant to pancreatic *β*-cell endoplasmic reticulum stress (Discussion), its direct role in diabetes remains unconfirmed, and we did not assess whether nearby variants act as eQTLs or pQTLs; accordingly, this signal is presented as hypothesis-generating rather than causal.

SNPs with low minor allele frequencies between 0.01 and 0.05 did not achieve high feature importance scores, particularly for atherosclerosis, myocardial infarction, and heart failure. This pattern may reflect the difficulty of learning stable attributions for lower-frequency variants in diseases with fewer cases, rather than the absence of biological relevance. Further investigation into low-frequency and rare variants, including their potential interactions with common variants, could provide additional insights into the genetic architecture of complex cardiometabolic traits [42].

### Transferability of risk prediction in diverse ancestries

DeepGP was trained in a European UKB population. To assess transferability, we evaluated performance in two additional UKB cohorts: African (*n = 3143*) and Caribbean (*n = 4243*). Supplementary Fig. 1 shows the principal component analysis (PCA) of the three cohorts. The European training and testing sets overlapped closely, indicating genetic similarity, whereas the Caribbean and especially the African cohorts were clearly separated from the European cohort, highlighting the challenge of transferability across ancestries.

Because of the limited sample sizes in the African and Caribbean cohorts, only type 2 diabetes had ¿500 cases in both ancestries. We therefore evaluated model performance for type 2 diabetes only. Given class imbalance in the testing sets, we used balanced metrics, including balanced accuracy [43], AUROC, area under the precision-recall curve (AUPRC), and Matthews correlation coefficient (MCC). Results are summarized in Table 2. This cross-ancestry analysis was restricted to DeepGP and LGBM. In initial analyses, the other deep learning comparators showed AUROC values close to 0.5, similar to PRS, whereas DeepGP and LGBM were the two informative models in this setting. The broader benchmark was performed in the European hold-out setting (Supplementary Fig. 4). Additional metrics, including precision, sensitivity, and specificity, are provided in Supplementary Table 4. The lower sensitivity observed for DeepGP at the default threshold highlights the need to select operating points in consultation with clinical stakeholders.

**Table 2:** Balanced evaluation metrics for type 2 diabetes risk prediction across African and Caribbean ancestries

Ancestry	Balanced accuracy		AUROC		AUPRC		MCC	
	DeepGP	LGBM	DeepGP	LGBM	DeepGP	LGBM	DeepGP	LGBM
African	**59.1**	53.6	**0.64**	0.54	**0.28**	0.20	**0.18**	0.05
Caribbean	**60.9**	53.1	**0.68**	0.56	**0.31**	0.22	**0.21**	0.05

PRS performed poorly in this cross-population setting and failed to provide meaningful risk stratification. In both African and Caribbean cohorts, PRS AUROC was close to random chance (0.5), indicating a substantial loss of performance, likely reflecting differences in genetic architecture between the European training population and the African and Caribbean test populations. In contrast, DeepGP improved AUROC by >0.1 relative to LGBM. These results support preliminary within-UKB evidence that DeepGP can retain predictive performance for type 2 diabetes in African and Caribbean ancestry subsets, but they should not be interpreted as external validation across diverse global populations. Performance was better in the Caribbean cohort (7/8 metrics), consistent with its slightly closer genetic distance to the European cohort in Supplementary Fig. 1.

## Discussion

In this study, we introduced DeepGP, a deep learning framework for predicting CMD risk from genomic and demographic data. Our findings support the potential of DeepGP as a complementary approach to genomic risk prediction, particularly for modelling complex genetic architectures and evaluating portability across ancestries. The present evidence for transferability is preliminary and restricted to UKB African and Caribbean ancestry subsets for type 2 diabetes. However, because the UKB predominantly comprises generally healthy participants of European ancestry aged 40 years or older, transferability was assessed without independent external cohorts, largely owing to the practical challenges of genomic data access. Future work will evaluate performance in independent cohorts with more diverse and admixed ancestries and, where feasible, under temporal and geographic splits. We will leverage biobank-scale resources such as FinnGen (Finland) [44] and All of Us (USA) [45] to further assess transferability.

The core component of DeepGP is the Bi-SSM module, motivated by recent sequence models for long-context learning. Unlike Enformer, Nucleotide Transformer, and Caduceus, which are primarily designed to learn representations from nucleotide sequences for molecular or regulatory prediction tasks [46–48], DeepGP operates on individual-level SNP dosage vectors for disease risk prediction and explicitly integrates demographic covariates. This design was selected after considering model complexity, memory constraints, and efficiency. In our initial experiments, conventional deep neural networks, including Dense and CNNs, did not show meaningful improvements over LGBM (Supplementary Fig. 4). We also investigated Transformer architectures and variants, but encountered memory bottlenecks on NVIDIA A10G GPUs (24 GB) because MHSA scales quadratically in time and memory with sequence length. Even a minimal Transformer baseline with one self-attention layer and hidden dimension 8 exceeded available GPU memory under the same SNP input representation; we therefore did not report Transformer AUROC because reducing sequence length further would compromise comparability. This result highlights the computational motivation for using Bi-SSM modules, which provide a more memory-feasible sequence-modelling approach for long SNP genotype sequences in the present setting. Direct benchmarking against optimized Transformer-based genomic models, such as Enformer, Nucleotide Transformer, or related long-sequence architectures, is not straightforward because these models are primarily designed for raw DNA sequence or nucleotide-token inputs, whereas DeepGP uses individual-level SNP genotype inputs. Adapting such models to matched SNP-level prediction tasks remains an important direction for future work. Even after LD clumping and splitting input SNPs across the 22 autosomal chromosomes, each chromosome-specific branch still contained up to *∼*10 000 SNPs. Mamba therefore emerged as the most practical choice, with linear complexity [34] and selective state space scanning. Using separate branches also avoids the memory growth that would arise from applying dense attention over the full genome-wide SNP sequence. We note that chromosome-specific branches increase the number of encoder parameters relative to a weight-shared design in which one Bi-SSM is reused across all chromosome branches. Weight sharing of this kind would reduce the parameter count while using similar or less memory, and is a reasonable alternative; we did not adopt it because chromosome-specific weights allow each branch to adapt to the differing length, SNP density, and LD structure of individual chromosomes. A further trade-off is that shared cross-chromosome sequence motifs are modelled only after chromosome-level pooling rather than by a single shared SNP encoder.

The bidirectional architecture further allows DeepGP to process sequences in both forward and reverse directions, capturing dependencies from upstream and downstream genomic context. Accordingly, DeepGP models long-range intra-chromosomal dependencies through the Bi-SSM encoder and integrates cross-chromosome information through an MHSA layer operating on chromosome-level embeddings, making it well suited to genome-scale risk prediction. We included the top six ancestry PCs to mitigate confounding from population stratification, consistent with PRS practice using a modest number of leading PCs [8]. We did not include disease-specific clinical covariates, such as BMI, HbA1c, or blood pressure, in order to maximize portability across datasets and avoid potential over-adjustment for intermediate phenotypes that may lie on the causal pathway for some outcomes.

Ablation analyses supported the contributions of multimodal input and sequence modelling (Supplementary Fig. 6). Across all variants, the covariate-only and SNP-only models showed the largest and second-largest reductions in mean AUROC relative to full DeepGP, respectively, indicating that the joint integration of genomic and demographic information is the primary contributor to predictive performance. Replacing the Bi-SSM encoder with a CNN produced the third-largest reduction, supporting the Bi-SSM architecture as an essential contributor when both input modalities are available. The additional reductions observed after adopting a unidirectional SSM or removing positional encoding further support the contributions of bidirectional contextual processing and positional information. Complementary controlled simulations, in which SNP heritability and the proportion of causal variants were held fixed, showed that both models improved as gene–gene and gene–environment interaction strength increased, while full DeepGP consistently outperformed the unidirectional SSM (Supplementary Fig. 2); these findings provide separate evidence that the models exploit nonlinear SNP signal and that bidirectional context contributes additional performance. A single un-split genome-wide encoder could not be trained within available GPU memory and was therefore not included in the ablation; we did not evaluate a weight-shared variant of the chromosome branches, nor did we separately ablate the multi-head self-attention aggregation layer. Future work should further dissect these components and extend the contextual ablations to ancestry-specific evaluation cohorts to assess whether the contribution of sequence modelling is retained across populations. Future work should also recompute attribution maps under Bi-SSM-ablated architectures and assess whether high-DeepLIFT, low-$\chi ^{2}$ variants are enriched in local regions containing SNPs with moderate GWAS evidence.

To further examine performance, we complemented the 1:1 case–control matched test sets used in prior studies [33] with additional analyses in imbalanced settings that preserve the observed prevalence of each CMD (Supplementary Table 2). We also benchmarked against PRS-CSx [39], a state-of-the-art multi-ancestry PRS method that infers posterior SNP effect sizes under coupled continuous-shrinkage priors across populations. For a fair comparison, PRS-CSx was trained on the same imbalanced training set, using a substantially larger cohort to increase statistical power. DeepGP achieved significantly higher AUROC within this natural-prevalence analysis (Supplementary Fig. 7; two-sided DeLong’s test, *P<.001*). We further computed 95% confidence intervals using a stratified paired bootstrap with 1000 replicates (Supplementary Table 5), and paired tests on bootstrap AUROC yielded consistent results (*P<.001*). The balanced and natural-prevalence analyses address related but distinct evaluation questions: the former directly compares DeepGP with conventional PRS and LGBM under matched 1:1 case–control sampling, whereas the latter directly compares DeepGP with PRS-CSx and PRS in larger cohorts that preserve the observed class distribution. Because PRS-CSx was not evaluated in the balanced analysis, AUROC values should not be compared numerically across the two settings. Differences in class distribution and training-set size may affect fitted models and performance estimates; the PRS-CSx result should therefore be interpreted as setting-specific evidence of incremental discrimination rather than universal superiority over advanced PRS methods. Although AUROC provides a threshold-independent measure of discrimination, clinically oriented evaluation in natural-prevalence settings requires additional metrics that are more sensitive to class imbalance, including AUPRC, positive predictive value, recall, and F1-score at clinically relevant decision thresholds. Future work should report these metrics systematically across diseases and benchmark methods to better assess potential clinical utility.

From a practical perspective, DeepGP is more computationally intensive to train than PRS or LGBM because it learns chromosome-level sequence encoders end to end; in our experiments, training required *∼*80 GPU hours. The trained model is nonetheless compact, comprising *∼*3.28 million parameters in its largest configuration. Single-sample inference, averaged over 50 timed runs after 10 warm-up iterations, required *∼*57.4 ms and 0.17 GiB of peak memory on an NVIDIA A10 GPU, compared with *∼*191.0 ms and 1.30 GiB for LightGBM under the same input dimensionality; on a single AMD EPYC CPU core (Zen 3), however, DeepGP inference was substantially slower (*∼*35.3 s, with 0.81 GiB of peak memory) than LightGBM (*∼*231 ms, 0.92 GiB). The higher cost of DeepGP is therefore concentrated in training and, at inference, in environments without GPU acceleration. This introduces a deployment trade-off: PRS and LGBM remain cheaper to train, simpler to implement, and efficient on CPU, whereas DeepGP achieves higher discrimination with fast, memory-efficient inference when a GPU is available and may be preferable when the objective is to capture nonlinear SNP-level context and maximize predictive performance. Thus, DeepGP may be most useful when improved discrimination justifies higher training cost and GPU-based deployment, for example, in research-scale biobank analyses or targeted risk-stratification workflows. These findings show that sequence-model-inspired methods can be applied to genome-wide analysis with modest GPU resources.

A further challenge in applying deep learning to genetics is the black-box nature of these models, which can obscure biologically meaningful insights [49]. To partly address this, we applied DeepLIFT to quantify the contribution of individual SNPs to disease prediction and rank nearby genes by attribution score. DeepLIFT, however, does not provide a calibrated false-positive rate for biological discovery, and attribution scores can be affected by correlated inputs, reference choice, and model uncertainty. Genes highlighted by attribution but not reaching genome-wide significance in GWAS should, therefore, be regarded as hypothesis-generating candidates requiring independent replication and functional validation. Establishing biological plausibility will require orthogonal evidence, including eQTL colocalization, pQTL analyses, tissue- or pathway-enrichment analyses, and functional studies where feasible. Future work should generate genome-wide DeepLIFT Manhattan plots to localize leading attribution signals and nominated genes across the genome. Further variant-level and gene-set annotation will be needed to determine whether prioritized variants fall within coding or regulatory regions, whether they produce nonsynonymous changes, and whether they are supported by eQTL, pQTL, or other functional evidence. Future work should also include GO/KEGG pathway enrichment and GTEx queries to assess whether DeepLIFT-prioritized genes show enrichment in relevant biological pathways or tissue-specific regulatory contexts.

Notwithstanding these limitations, several of the genes prioritized by DeepLIFT have functions with plausible relevance to cardiometabolic biology, which supports their interpretation as hypothesis-generating candidates. For type 2 diabetes, alongside the well-established *TCF7L2* locus, the highly ranked *PDIA5* encodes a protein disulfide isomerase that facilitates ATF6*α*-mediated endoplasmic reticulum stress signalling [50], a pathway implicated in pancreatic *β*-cell dysfunction [51], while *BHLHE40* (also known as *DEC1*) is a circadian transcriptional regulator that has been linked to *β*-cell maturation and glucose-stimulated insulin secretion [52]. For atrial fibrillation, the leading attribution signal corresponded to *PITX2* at the well-characterized 4q25 locus [40], and for myocardial infarction the prioritized genes included *KCNE4*, a potassium-channel regulatory subunit abundantly expressed in ventricular myocardium [53]. These correspondences rest on prior knowledge of gene function rather than the formal colocalization, eQTL/pQTL, or pathway-enrichment analyses outlined above, and should not be interpreted as evidence of causality.

Several limitations remain. GWAS and LD clumping were performed once on the European training set before five-fold cross-validation for hyperparameter selection, rather than repeated within each fold. Although the final hold-out test sets were never used for feature selection, this design may slightly overestimate internal cross-validation performance. LD clumping may discard functional variants that are highly correlated with tag SNPs, and standard PCA may not fully capture fine-scale population structure across ancestries. Because only LD-clumped tag SNPs were provided to the model, Bi-SSM branches did not directly access nearby variants that were excluded from the disease-specific SNP feature sets; information from omitted variants can only be indirectly represented through LD with retained tag SNPs. Because disease-specific SNP feature sets were derived using GWAS-based LD clumping, the current framework remains partly dependent on the initial feature-selection step. The same SNP sets were used across DeepGP and all baseline models, reducing the likelihood that DeepGP’s comparative performance was driven by a unique feature-selection advantage. Nevertheless, future real-data robustness analyses should evaluate how predictive performance changes when weakly associated or random SNPs are added to the disease-specific feature sets. We also did not model environmental exposures such as smoking, diet, physical activity, or medications. Future work will evaluate LD-aware feature selection and fine-mapping-guided strategies to preserve multiple causal signals in high-LD regions [39], adopt ancestry-specific PCA with projection from more diverse reference panels, and incorporate additional covariates for disease-specific deployment, such as blood pressure for coronary artery disease risk [54]. As GPU memory and bandwidth improve, DeepGP will be able to ingest more comprehensive genetic data, and joint genome-wide models that retain a broader spectrum of variants may further improve prediction and yield endogenous etiological insights into CMDs [55]. In addition, our simulator is necessarily a simplification of CMD genetic architecture. As more appropriate tools become available, we will evaluate DeepGP under richer, ancestry-aware simulation and phenotype-generation frameworks that better reflect polygenicity, pleiotropy, rare variation, and realistic LD, and complement these analyses with epistasis-aware or empirically anchored simulations and, where power permits, large external cohorts designed for interaction testing. Finally, because DeepGP is modular in its input branches, a natural extension is to integrate additional molecular modalities, such as transcriptomic, proteomic, or epigenomic data, alongside genotypes to capture complementary biological signals for cardiometabolic risk prediction.

## Methods

### Data preprocessing

To reduce the dimensionality of the input data and mitigate the effect of LD, we processed the QC’ed SNPs using LD clumping, performed exclusively on the European training set after excluding the fixed hold-out test set, to prevent leakage into the hold-out test set. This procedure consisted of two steps. First, we conducted a GWAS on the European training set after excluding the fixed hold-out test set, following the standard pipeline using PLINK v2.00 to estimate the association between each SNP and each CMD [33]. Second, we performed LD clumping using relatively inclusive thresholds (–clump-p1 0.05, –clump-p2 0.05, –clump-r2 0.7, –clump-kb 500) based on the *P*-values derived from the GWAS. This inclusive threshold was chosen to retain a larger number of potentially informative variants for the deep learning model. The LD clumping procedure selected a tag SNP with the smallest *P*-value within each LD group. As a result, each CMD had a unique list of variants. Variants not selected by LD clumping were not used as model inputs, and therefore nearby excluded SNPs were not directly available to the Bi-SSM branches. On average, the dimensionality of SNPs was reduced from 13 277 480 to *∼*100 000 for each disease, with the number of variants varying from 10 000 to 2000 across chromosomes. This reduction in dimensionality not only simplifies the computational complexity of the analysis but also enables the model to focus on the most informative SNPs while accounting for the redundancy introduced by LD. CMD phenotypes were defined using UKB multi-source disease definitions integrating self-report, hospital records, procedural codes, laboratory measures, and death registry data; full code definitions are provided in Supplementary information.

The dosage value of the minor allele for each SNP varies within the range of [0, 2], where 0 represents the absence of the minor allele, 1 represents the presence of one copy of the minor allele (heterozygous), and 2 represents the presence of two copies of the minor allele (homozygous). The value embedding was performed using a 1D convolutional layer. The positional information of the SNPs on each chromosome was obtained and converted to absolute position encodings using sine and cosine functions for geometric progression, as proposed in the vanilla Transformer architecture [56]. Finally, together with the covariates, the input features were normalized using standard scaling to ensure that the covariates have zero mean and unit variance, which improves the convergence and stability of the deep learning model during training.

### Model architecture of DeepGP

DeepGP is a multimodal deep learning model designed to estimate the risk probability of CMDs using input data consisting of SNPs and covariates. The model architecture incorporates 22 Bi-SSM branches, each processing the SNP data from one of the 22 autosomal chromosomes. We process variants per chromosome to model long-range statistical dependencies within chromosomes while maintaining computational scalability. This architecture balances biological plausibility, given the enrichment of cis structure in the genome, with computational feasibility at genome scale. We do not interpret these dependencies as evidence of epistasis. In addition, DeepGP includes an FCN module that processes the covariates, which allows the model to consider the contributions of environmental factors to the overall disease risk. This multi-input architecture enables DeepGP to effectively harness the information from multimodal data and provide more accurate and personalized risk predictions for CMDs.

The Bi-SSM branches are specialized in capturing the complex, nonlinear relationships between the genetic variants and the disease risk. On each branch, the first step is to process SNP dosages via an embedding layer to obtain the desired dimension (*N*) of hidden states and add positional encoding. The core idea is a structured state space sequence model that maps an input sequence *x(t)*, i.e. embedding representation of SNP *x* on position *t* for the first layer, to an output sequence *y(t)* with an intermediate hidden state representation *h*. The model is constructed with system parameters $\mathbf{A}$, $\mathbf{B}$, and $\mathbf{C}$ as follows:


(1) \begin{eqnarray*}& h^{\prime}(t) = \mathbf{A}h(t) + \mathbf{B}x(t), \quad y(t) = \mathbf{C}h(t),\end{eqnarray*}

where $\mathbf{A} \in \mathbb{R}^{N \times N}$ is the state matrix for evolution parameters, and $\mathbf{B} \in \mathbb{R}^{N \times 1}$ and $\mathbf{C} \in \mathbb{R}^{1 \times N}$ represent the projection parameters. The state matrix $\mathbf{A}$ governs the transition dynamics of the hidden states, while the projection matrices $\mathbf{B}$ and $\mathbf{C}$ map the input sequence and hidden states to the appropriate dimensions. In practice, the discrete form of these continuous parameters can be denoted as follows with zero-order hold transformation


(2) \begin{eqnarray*}& \bar{\mathbf{A}}=\exp(\Delta\mathbf{A}), \quad \bar{\mathbf{B}}=\mathbf{A}^{-1}(\exp(\Delta\mathbf{A})-\mathbf{I})\mathbf{B}_{t}\end{eqnarray*}

where *Δ* is a time scaler parameter, i.e. step size, and therefore Equation ( 1) can be rewritten as follows:


(3) \begin{eqnarray*}& h^{\prime}_{t+1} = \bar{\mathbf{A}}h_{t} + \bar{\mathbf{B}}x_{t}, \quad y_{t+1} = \mathbf{C}h_{t}.\end{eqnarray*}

To enable the model to attend to specific inputs, Mamba introduces a selection mechanism that projects parameters $\mathbf{B}$ and $\mathbf{C}$ via linear transformations on $x_{t}$, and uses *Δ*, which also linearly projects $x_{t}$ via a linear transformation followed by a nonlinear activation function. In the Bi-SSM module, we utilize two Mamba modules to process hidden states in both forward and backward directions and concatenate them together at the output. Combining the covariate representation $y_{\mathrm{cov}}$ from the FCN the output of DeepGP is denoted as follows:


(4) \begin{eqnarray*}& y_{o} = \mathbf{Opt}(\mathbf{Concat}(\mathbf{Pooling}(\mathbf{Proj}(y_{\mathrm{forward}} + y_{\mathrm{backward}}))^{1\dotsc 22}, y_{\mathrm{cov}})),\end{eqnarray*}

where $\mathbf{Opt}$ represents the output head consisting of an MHSA module followed by an FCN, $\mathbf{Concat}$ performs concatenation of features, $\mathbf{Proj}$ applies a projection transformation, and $\mathbf{Pooling}$ aggregates the projected bidirectional features from the combined forward ($y_{\mathrm{forward}}$) and backward ($y_{\mathrm{backward}}$) representations, which are then combined with covariate features ($y_{\mathrm{cov}}$). $y_{o}$ is a risk probability processed by a sigmoid layer for binary classification. FCN modules were chosen for covariate processing and the prediction head to preserve end-to-end differentiability, while MHSA complements Bi-SSM by modelling long-range genomic dependencies and heterogeneity in chromosome length.

### Model training and evaluation

To address the highly imbalanced ratio of cases to controls for the eight selected CMDs in the UKB, which ranges from 2.5% (stroke) to 9.8% (coronary artery disease), we constructed a balanced development dataset for each disease by ensuring an equal number of cases and controls. This approach ensures that the model is trained on an equal representation of both classes, mitigating potential bias towards the majority class.

From the balanced development set, we randomly allocated 15% of the cases and controls to create a hold-out testing set for unbiased performance evaluation. The remaining samples were assigned to the training set. To prevent overfitting and select model hyperparameters, we employed five-fold cross-validation on the training set. The training data were partitioned into five equal folds; in each iteration, one-fold served as the validation set and the remaining four as the training set. Hyperparameters were chosen to maximize the mean AUROC across the five-folds. Because LD clumping yields disease-specific SNP feature sets, validation and tuning were performed separately for each disease. The hyperparameter ranges and final configuration are provided in Supplementary Tables 6 and 7, respectively. All reported results are from fixed, ancestry-specific hold-out testing sets that were not used for training or hyperparameter tuning. The full development pipeline is illustrated in Supplementary Fig. 8.

For classification tasks, we employed the binary cross-entropy loss function to optimize the model parameters, which measures the dissimilarity between the predicted probabilities and the true binary labels. To optimize the model parameters, we used the Adam optimizer, which adapts the learning rate for each parameter based on its historical gradients. Additionally, we incorporated early stopping and L2 regularization with a weight decay parameter in the Adam optimizer to further reduce the risk of overfitting.

PRS was derived from in-house GWAS summary statistics generated from the training set (Fig. 3a) via a standard protocol [6], using the same LD-clumped SNP set as the other models. SNP effect sizes were converted to individual scores using PLINK. For the standard PRS baseline, the LR inputs were the participant-level PRS together with age, sex, and the first six ancestry PCs. The PRS-CSx baseline similarly combined its posterior-effect PRS with age, sex, and the same six PCs. DeepGP and the SNP-level baseline models received the LD-clumped SNP dosage vector together with these covariates. Thus, DeepGP did not have exclusive access to demographic information in any baseline comparison. All models were implemented in Python. Deep learning models, including DeepGP, CNN, and Dense (Supplementary Fig. 4), were developed in PyTorch and PyTorch Lightning, whereas machine learning baselines were implemented using LightGBM and scikit-learn. All models were benchmarked using an identical five-fold cross-validation and hyperparameter optimization framework. For internal cross-validation, SNP feature selection was not repeated inside each validation fold; the implications of this design are addressed in the Discussion. DeepGP was trainable on modest GPU resources and supported inference on a single GPU or CPU. Full implementation details, software versions, hardware specifications, and baseline construction procedures are provided in the Supplementary Information.

### Phenotype simulation

To evaluate the performance and robustness of DeepGP and the baseline methods, we conducted validation experiments using simulated phenotypes. These phenotypes were generated by a simulator that incorporates complex biological mechanisms, including intricate genetic architectures, gene–gene interactions (i.e. epistasis), and gene–environment interactions. This approach allows for a controlled assessment of the models’ ability to capture and predict complex trait outcomes under various scenarios that closely mimic real-world genetic complexity. Following the type 2 diabetes analysis setup, we used only European training and testing cohorts, incorporating SNPs from all autosomal chromosomes. We used the original genotype data from our preprocessing pipeline and sampled causal SNPs from a mixture of normal distributions, comprising 1% of the total number of SNPs, which reflects the complex genetic architecture of many traits. Given $N_{s}$ sampled SNPs, the effect size variances were allocated as follows: large-effect SNPs ($0.2h^{2}_{\mathrm{snp}}/0.02N_{s}$), moderate-effect SNPs ($0.2h^{2}_{\mathrm{snp}}/0.05N_{s}$), and small-effect SNPs ($0.6h^{2}_{\mathrm{snp}}/0.93N_{s}$), ensuring that 2%, 5%, and 93% of SNPs contribute to the total SNP heritability ($h^{2}_{\mathrm{snp}}$), respectively [57].

We implemented a two-step process to incorporate complex genetic and environmental interactions. First, we randomly selected SNPs from the group with large effect sizes to model gene–gene interactions. For each selected SNP, we formulated a gene–gene interaction matrix and multiplied it with interaction effects, following an established approach for simulating epistatic effects [58]. Second, we introduced gene–environment interactions by modelling the target trait as a nonlinear function of a heritable environmental factor [59]. The environmental factor was calculated as the product of the genotype matrix and a set of random SNP effects, plus residuals. This approach simulates a realistic scenario where genetic factors influence susceptibility to environmental exposures.

Finally, we rescaled the overall residuals to standardize the trait distribution. To generate binary traits, we transformed the continuous phenotypes into probabilities using a logistic sigmoid function. These probabilities were then used to sample from a binomial distribution, introducing an additional randomness to mimic the stochastic nature of disease occurrence. By incorporating these additional steps, we created a more realistic and challenging dataset for evaluating the predictive performance of DeepGP and baseline methods across a range of phenotypic distributions and outcome types.

### Interpretability analysis

We used DeepLIFT [36] to interpret DeepGP and identify SNPs contributing most strongly to risk prediction. DeepLIFT was selected because it provides efficient feature attribution and was substantially more memory-efficient than SHAP [60] for our model and data scale.

For an input *x* and reference input $\bar{x}$, DeepLIFT assigns an importance score $S_{\Delta x_{i} \Delta y}$ to feature *i*:


(5) \begin{eqnarray*}& S_{\Delta x_{i} \Delta y} = \sum_{j} m_{ij} \Delta y_{j},\end{eqnarray*}

where $m_{ij}$ is the multiplier relating the change in neuron *j* to the output change, and $\Delta y_{j} = y_{j} - \bar{y}_{j}$. The feature difference is $\Delta x_{i} = x_{i} - \bar{x}_{i}$.

Multipliers are propagated recursively through the network:


(6) \begin{eqnarray*}& m_{ik} = \sum_{j} m_{ij} m_{jk},\end{eqnarray*}

where $m_{ik}$ denotes the total contribution of feature *i* to the final output through intermediate neurons. We applied DeepLIFT to the trained DeepGP model to obtain SNP-level importance scores. The reference input was set to the mean value of each SNP across all samples, which was zero after standardization. We then compared these importance scores with the Chi-square statistics from GWAS to assess concordance between model attribution and statistical association. A glossary of key terms and abbreviations is provided in Supplementary Table 8.

**Key Points**

- DeepGP (Deep learning-based Genome-wide Predictor) integrates genome-wide variants and covariates in a state space deep learning architecture for cardiometabolic disease (CMD) risk prediction.
- DeepGP outperformed polygenic risk score and LightGBM in simulations and across eight UK Biobank (UKB) CMDs.
- DeepLIFT analysis recovered established risk genes and nominated additional hypothesis-generating loci beyond conventional genome-wide association studies signals.
- The model retained preliminary within-UKB predictive performance in African and Caribbean ancestry cohorts for type 2 diabetes.
- State space models may improve genomic risk stratification for complex diseases and merit further evaluation of cross-ancestry transferability in independent, more diverse external cohorts.

## Supplementary Material

supplementary_bbag550

## Data Availability

The UKB is an open access resource available to approved researchers. Access to the data used in this study is available through an application procedure described at https://www.ukbiobank.ac.uk/enable-your-research. Because the UKB data-use agreement prohibits redistribution of individual-level genotype and phenotype data, development and test datasets cannot be deposited publicly. To support reproducibility, the GitHub repository provides the DeepGP source code, preprocessing scripts, model configuration files, and instructions for recreating the PLINK-based GWAS, LD clumping, training, and evaluation pipeline after obtaining UKB access. Trained model weights and derived feature lists can be shared with researchers who hold the required UKB approval and satisfy applicable data-transfer conditions. The code implementation of DeepGP is available on GitHub (https://github.com/tndrg/DeepGP).
