# Supplementary material for: Multimodal deep learning enhances genomic risk prediction for cardiometabolic diseases
Source: Brief Bioinform. 2026 Oct 8;27(5):bbag550. doi: 10.1093/bib/bbag550 (PMC13647273; doi:10.1093/bib/bbag550)
Supplement: Supplementary file 1 [file supplementary_bbag550.pdf]

Supplementary information: Multimodal deep learning enhances genomic risk prediction for cardiometabolic diseases

Taiyu Zhu*, Upamanyu Ghose, Héctor Climente-González, Joanna Howson, Sile Hu and Alejo Nevado-Holgado

Details of Data Characteristics and Ancestries

Tables 1 and 2 present, respectively, the numbers of samples in the balanced and imbalanced (natural prevalence) training and testing sets for each cardiometabolic disease, along with the total number of cases. These samples were obtained after filtering to include only unrelated European participants based on the UKB's quality control procedures. Disease classifications were derived using the multiple-source data definition approach within the UKB's defined disease phenotypes, which integrates information from ICD codes, self-reports, hospital records, and other clinical data sources to ensure robust phenotyping. Fig. 1 shows the PCA visualization of the African and Caribbean ancestry validation cohorts within the UKB.

Supplementary Table 1: Sample sizes of the balanced training and testing sets for the considered CMDs in the UK Biobank

Phenotype	Training set (cases)	Testing set (cases)
Atherosclerosis	60,678 (30,339)	10,708 (5,354)
Atrial fibrillation	30,862 (15,431)	5,446 (2,723)
Cardiovascular disease	54,672 (27,336)	9648 (4,824)
Coronary artery disease	60,266 (30,133)	10,636 (5,318)
Heart failure	20,634 (10,317)	3,642 (1,821)
Myocardial infarction	24,714 (12,357)	4,360 (2,180)
Stroke	16,004 (8,002)	2,824 (1,412)
Type 2 diabetes	44,828 (22,414)	7,912 (3,956)

Supplementary Table 2: Sample sizes of the imbalanced training and testing sets for the considered CMDs in the UK Biobank

Phenotype	Training set (cases)	Testing set (cases)
Atherosclerosis	258,106 (30,339)	45,549 (5,354)
Atrial fibrillation	301,220 (15,431)	53,157 (2,723)
Cardiovascular disease	301,014 (27,336)	53,121 (4,824)
Coronary artery disease	309,832 (30,133)	54,677 (5,318)
Heart failure	301,220 (10,317)	53,157 (1,821)
Myocardial infarction	309,832 (12,356)	54,677 (2,181)
Stroke	309,832 (8,002)	54,677 (1,412)
Type 2 diabetes	309,830 (22,414)	54,676 (3,956)

Simulation Analysis of Nonlinear Genetic Effects

To more directly isolate the contribution of bidirectional sequence modelling to nonlinear genetic effects, we performed a controlled simulation in which SNP heritability ($h^2_{snp} = 0.30$) and the proportion of causal variants were held fixed

*Corresponding author: taiyu.zhu@psych.ox.ac.uk

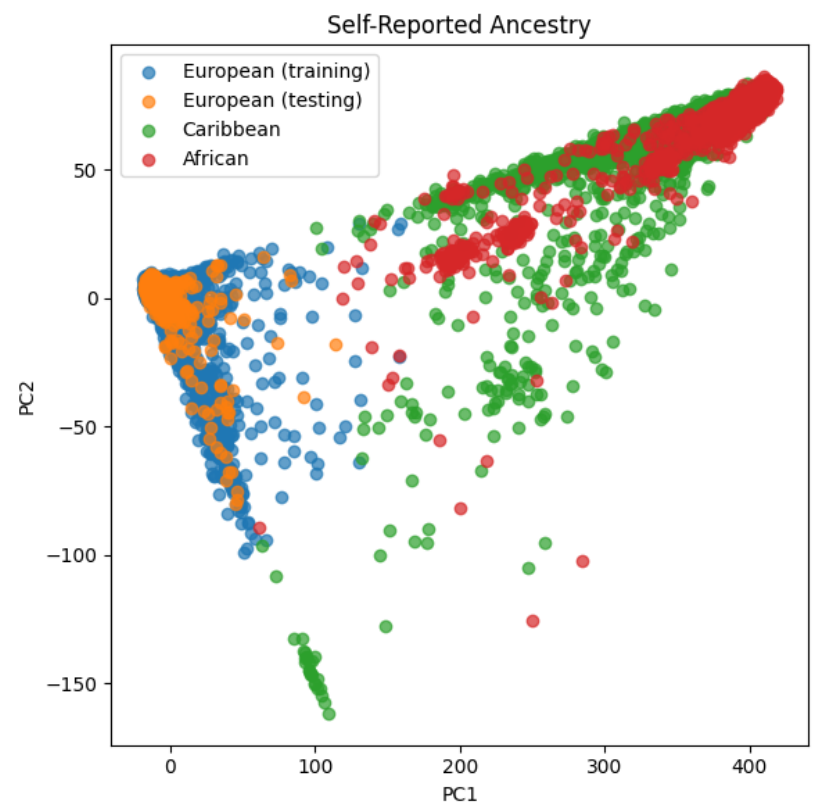

Supplementary Figure 1: Principal component analysis to visualize the three ancestries in the UK Biobank. Two major principal components (PC1 and PC2) are used, where the colour of each point represents the group of data set.

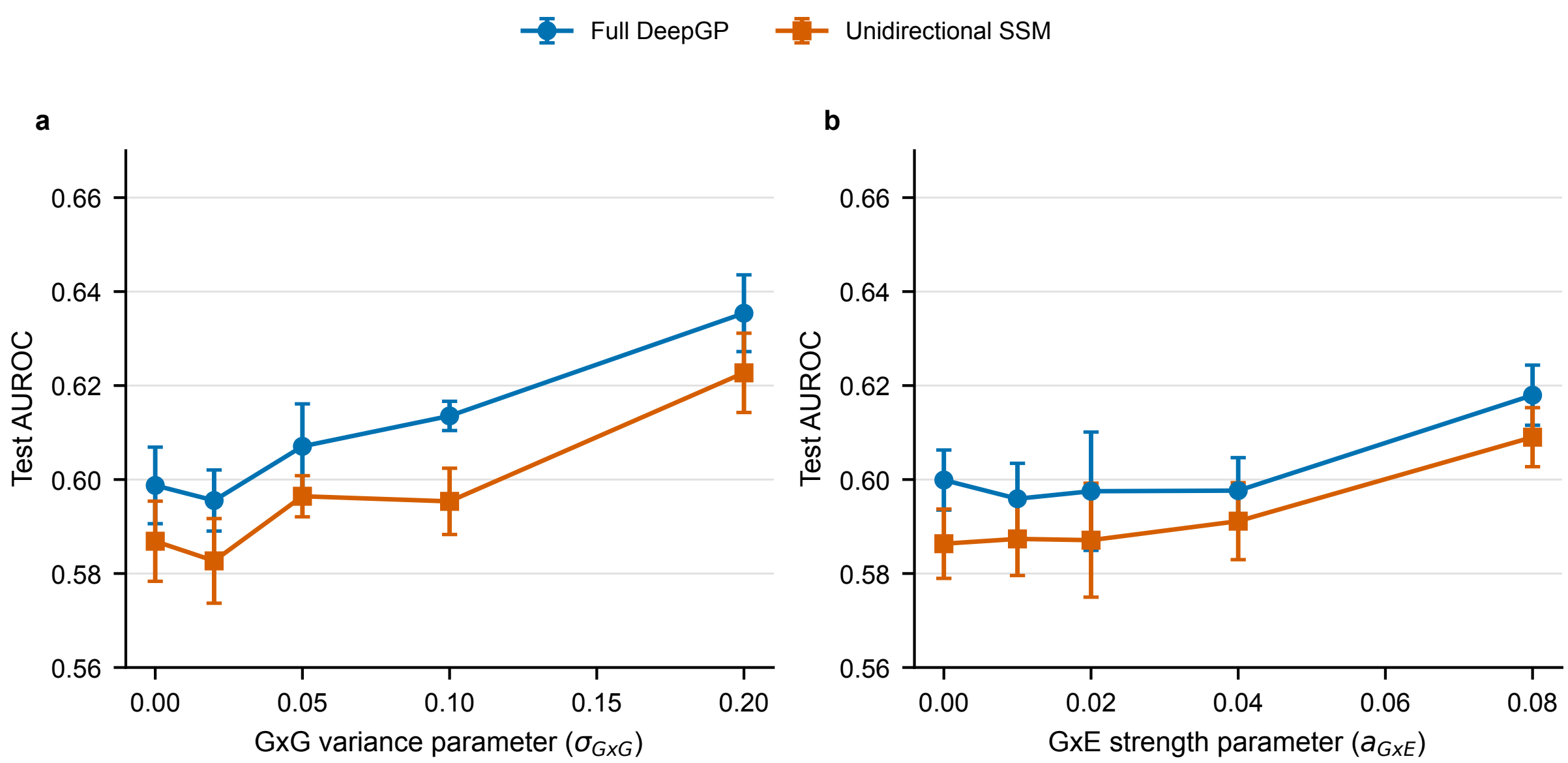

Supplementary Figure 2: Controlled simulation across increasing nonlinear interaction strength. (**a**) Test AUROC as the gene–gene interaction variance parameter (σ_{GxG}) is increased while SNP heritability and the proportion of causal variants are held fixed. (**b**) Test AUROC as the strength of the nonlinear gene–environment term (a_{GxE}) is increased under the same fixed settings. Total continuous phenotype variance is maintained at approximately 1 by adjusting residual variance. Points show the mean across 10 simulation replicates and error bars show 95% confidence intervals. Full DeepGP is compared with a unidirectional SSM to assess the contribution of bidirectional contextual processing.

while the strengths of gene–gene and gene–environment interactions were varied separately (Fig. 2). Full DeepGP was compared with a unidirectional SSM, which reduces the contextual capacity of the sequence encoder, using identical simulated datasets with 10 replicates per setting. Because the simulator maintains total continuous phenotype variance at approximately 1 by reducing residual variance as interaction variance increases, stronger interactions replace residual noise with structured nonlinear signal rather than increasing total variance. Full DeepGP consistently outperformed the unidirectional SSM across all interaction strengths, and both models improved as the gene–gene interaction variance and the gene–environment interaction strength increased. The advantage of the bidirectional model was modest and remained approximately constant rather than widening with interaction strength, which is expected because the unidirectional control still accesses substantial sequence context.

Fig. 3 provides a simulation-based diagnostic comparison between DeepLIFT attribution scores and marginal χ^2 statistics. Additive-dominant causal SNPs tend to show higher χ^2 statistics, whereas interaction-involved causal SNPs can receive high DeepLIFT attribution despite weaker marginal association. This analysis illustrates why model

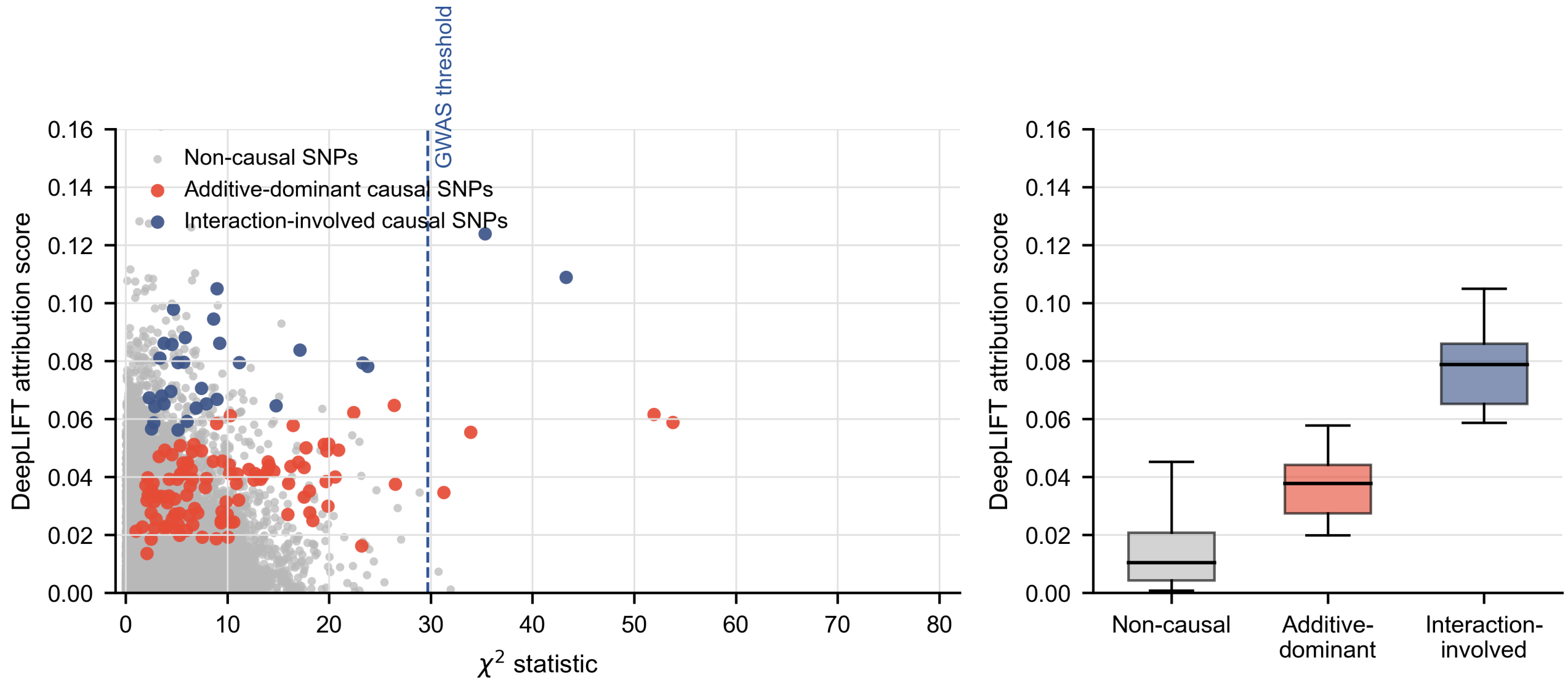

Supplementary Figure 3: Simulation-based comparison between DeepLIFT attribution scores and marginal χ^2 statistics. Left: SNP-level scatter plot comparing χ^2 statistics and DeepLIFT attribution scores, with non-causal, additive-dominant causal, and interaction-involved causal SNPs shown separately. The dashed vertical line indicates the approximate genome-wide-significance χ^2 threshold. Right: distribution of DeepLIFT attribution scores by simulated SNP class.

attribution and GWAS association statistics can highlight partially distinct variant sets.

Comparative Performance Analysis

Table 3 provides additional evaluation metrics for genomic risk prediction across eight CMDs in the balanced European ancestry population, while Table 4 presents supplementary metrics for validation on African and Caribbean ancestries specifically for type 2 diabetes. The value of the metrics shown in Supplementary Tables 3 and 4 are dependent on the decision threshold being chosen for DeepGP, as it is the case for most machine learning methods. For instance, a lower decision threshold would increase the sensitivity and decrease the specificity, while a higher decision threshold would do the opposite. Which is the most convenient threshold therefore depends on the intended application. Threshold-independent and balanced metrics, which do not depend on such threshold, are shown in Tables 1 and 2 to summarize overall performance.

Fig. 4 in Supplementary illustrates the AUROC curves of various traditional machine learning and deep learning methods in genomic risk prediction for the considered CMDs. We report relative reduction in misclassified cases (error rate) versus a baseline as

$$\frac{(\mathrm{Err}_{\mathrm{baseline}} - \mathrm{Err}_{\mathrm{model}})}{\mathrm{Err}_{\mathrm{baseline}}} \times 100\% \tag{1}$$

where Err=1-Accuracy.

Concordance Between DeepLIFT Attributions and GWAS Statistics

Fig. 5 examines how the rank concordance between DeepLIFT attribution scores and marginal χ^2 statistics depends on the GWAS p-value threshold used to define the SNP set, using coronary artery disease as a representative example. Concordance, measured by Spearman's ρ, increased monotonically as the threshold became more stringent, rising from $\rho \approx 0.03$ when all SNPs with $p < 0.05$ were included to $\rho = 0.71$ at the genome-wide-significance threshold ($p < 5 \times 10^{-8}$). This indicates that model attribution and marginal association agree most strongly among robustly associated loci, while diverging for weakly associated variants, consistent with DeepLIFT capturing multivariable and interaction contributions in addition to marginal effects.

Ablation Analysis of Model Components

Fig. 6 summarizes ablation analyses evaluating the relative contribution of model input modalities and architectural

Supplementary Table 3: Performance of genomic risk prediction on hold-out testing data of European ancestry

Phenotype	Method	ACC	Prec	Sens	Spec	AUROC	F1
Atherosclerosis	DeepGP	**68.6**	**67.1**	**72.8**	**64.3**	**0.75**	**0.70**
	LGBM	64.7	64.3	66.4	63.2	0.70	0.66
	PRS	65.7	64.2	70.7	60.7	0.70	0.67
Atrial fibrillation	DeepGP	**67.6**	**68.9**	64.1	**71.1**	**0.74**	**0.68**
	LGBM	65.7	65.0	68.2	63.3	0.71	0.66
	PRS	65.9	64.1	**72.2**	59.6	0.70	0.67
Cardiovascular disease	DeepGP	**67.1**	**67.5**	65.9	**68.3**	**0.74**	**0.67**
	LGBM	63.8	63.4	65.6	62.1	0.69	0.64
	PRS	64.4	62.5	**72.1**	56.8	0.68	0.66
Coronary artery disease	DeepGP	**67.4**	**67.5**	66.9	**67.8**	**0.74**	**0.67**
	LGBM	63.9	63.4	65.8	62.0	0.68	0.64
	PRS	63.5	62.0	**69.9**	57.2	0.69	0.65
Heart failure	DeepGP	**67.1**	**64.0**	**78.3**	55.9	**0.72**	**0.70**
	LGBM	62.8	62.8	62.6	**62.9**	0.68	0.63
	PRS	62.7	62.0	66.3	59.2	0.66	0.65
Myocardial infarction	DeepGP	**69.0**	**72.0**	62.3	**75.7**	**0.76**	**0.67**
	LGBM	65.8	65.1	68.2	63.5	0.71	0.66
	PRS	62.9	61.6	**68.3**	57.3	0.67	0.64
Stroke	DeepGP	**63.2**	**62.0**	**68.2**	**58.3**	**0.68**	**0.65**
	LGBM	59.6	59.5	59.8	59.3	0.62	0.60
	PRS	57.9	57.5	60.8	55.1	0.60	0.60
Type 2 diabetes	DeepGP	**60.4**	**59.5**	**65.5**	55.5	**0.64**	**0.62**
	LGBM	59.5	59.2	59.6	**58.9**	0.61	0.59
	PRS	57.7	57.4	59.8	55.7	0.60	0.59

Supplementary Table 4: Performance of genomic risk prediction for type 2 diabetes on African and Caribbean ancestries

Method	Phenotype	ACC	Prec	Sens	Spec	AUROC	F1
DeepGP	African	**76.9**	**31.5**	32.3	**85.9**	**0.64**	**0.32**
	Caribbean	**76.1**	**34.3**	37.4	**84.5**	**0.68**	**0.36**
LGBM	African	53.7	18.9	**53.6**	53.7	0.54	0.28
	Caribbean	54.9	19.8	**50.3**	55.9	0.56	0.28

components. Full DeepGP achieved the highest mean AUROC across the eight CMDs. Relative to full DeepGP, the covariate-only and SNP-only variants produced the largest and second-largest reductions in mean AUROC, respectively, indicating that joint integration of genomic and demographic information is the primary contributor to predictive performance. Replacing the Bi-SSM encoder with a CNN produced the third-largest reduction, supporting Bi-SSM as an essential architectural contributor. The additional reductions observed with a unidirectional SSM and without positional encoding further support the contributions of bidirectional contextual processing and positional information.

Model Performance on Real-World Prevalence Datasets

Fig. 7 shows AUROC for DeepGP, PRS-CSx, and PRS trained and evaluated under imbalanced (natural-prevalence) splits (see Supplementary Table 2 for sample sizes). For each model–cohort combination, 95% confidence intervals were obtained via a stratified, paired bootstrap with 1,000 replicates; detailed intervals are reported in Table 5. Pairwise comparisons between DeepGP and each baseline were assessed with DeLong's test and were significant in all cohorts (two-sided $p < 0.001$). As a robustness check, paired tests on the bootstrap replicate AUCs reached the same conclusion ($p < 0.001$).

Model Hyperparameters and Development Pipeline

Table 6 presents the hyperparameter ranges evaluated during model validation. For each of the considered CMDs, the final hyperparameter configuration, as shown in Table 7, was selected based on the best AUROC performance, resulting

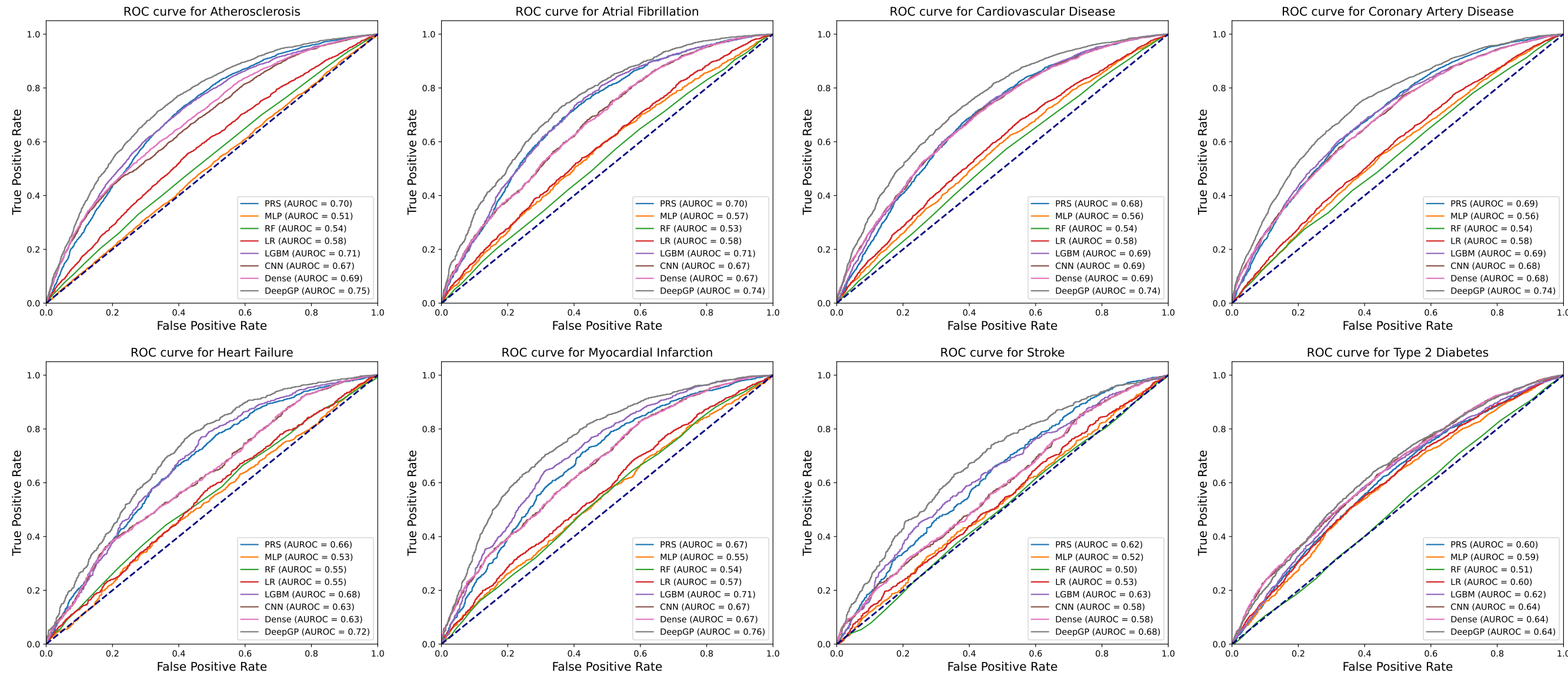

Supplementary Figure 4: Predictive performance across all the considered methods. AUROC for DeepGP and baseline methods: PRS, multilayer perceptron (MLP), random forest (RF), logistic regression (LR), LGBM, convolutional neural network (CNN), and dense neural network (Dense).

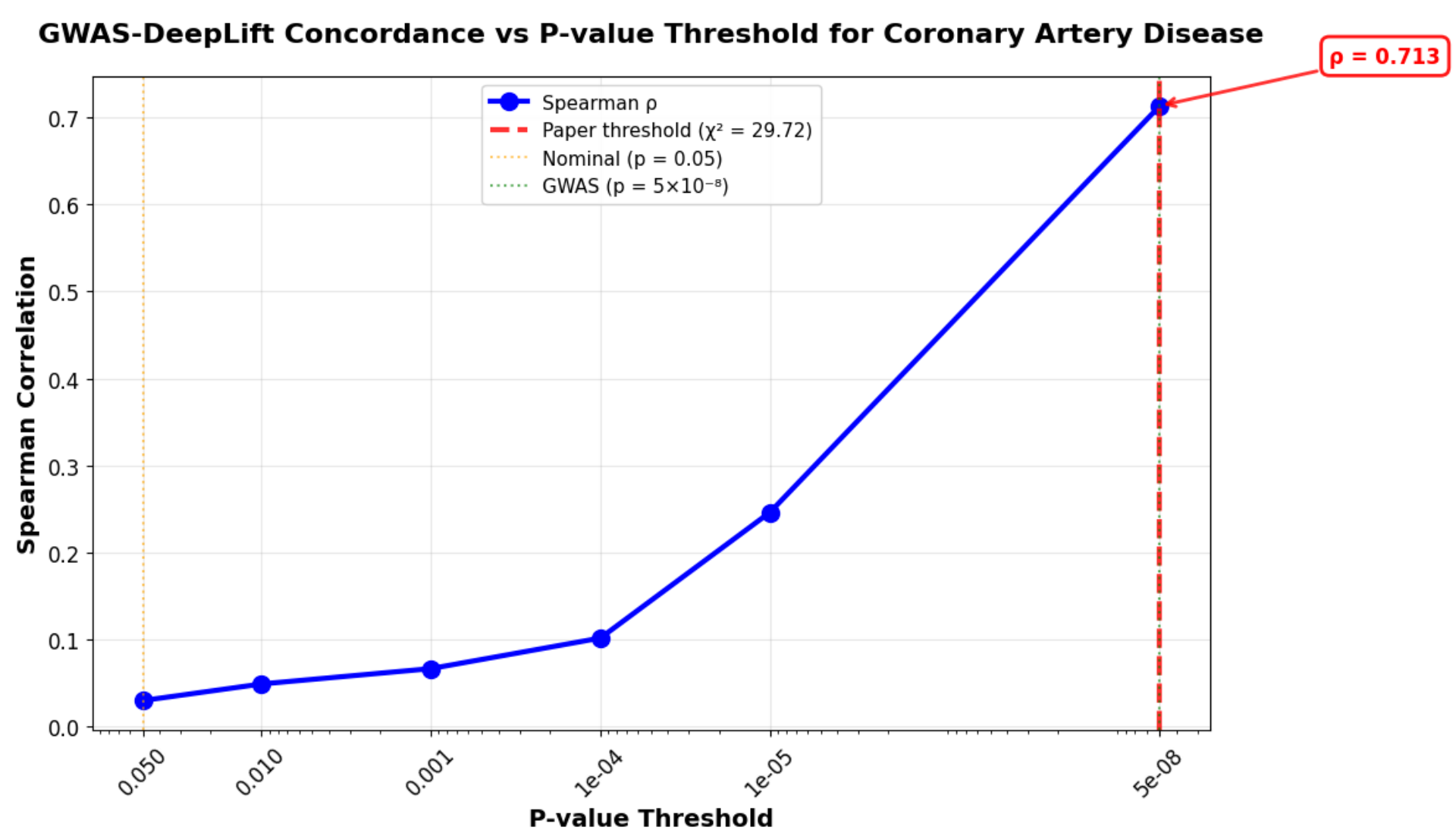

Supplementary Figure 5: Threshold-dependent concordance between DeepLIFT attribution scores and GWAS χ^2 statistics for coronary artery disease. Spearman rank correlation (ρ) between SNP-level DeepLIFT attribution scores and χ^2 statistics, computed over the SNPs passing each GWAS p-value threshold. Concordance increases as the threshold becomes more stringent, reaching $\rho = 0.71$ at the genome-wide-significance threshold. The red dashed vertical line marks the genome-wide-significance threshold ($\chi^2 = 29.72$, equivalently $p = 5 \times 10^{-8}$), and the orange dotted vertical line marks the nominal threshold ($p = 0.05$).

in slight variations in the optimal model parameters across different diseases. These disease-specific optimizations ensure that each model is tailored to capture the unique genetic architecture and risk factors associated with each particular condition. Hyperparameters were selected using the five-fold cross-validation pipeline shown in Fig. 8; final results are reported on fixed, independent hold-out cohorts.

All models in this study were implemented using Python 3.10. The deep learning models, including DeepGP, CNN and Dense shown in Supplementary Fig. 4, were developed using PyTorch 2.1.1. We leveraged PyTorch Lightning 2.0.8 to accelerate training and enable efficient utilization of multiple GPUs. All deep learning models were trained on NVIDIA A10 Tensor Core GPUs with CUDA 11.8. DeepGP was trained for approximately 10 hours on an AWS EC2 G5 instance with 8×A10 GPUs, corresponding to 80 GPU hours. Clinical inference requires only forward passes and

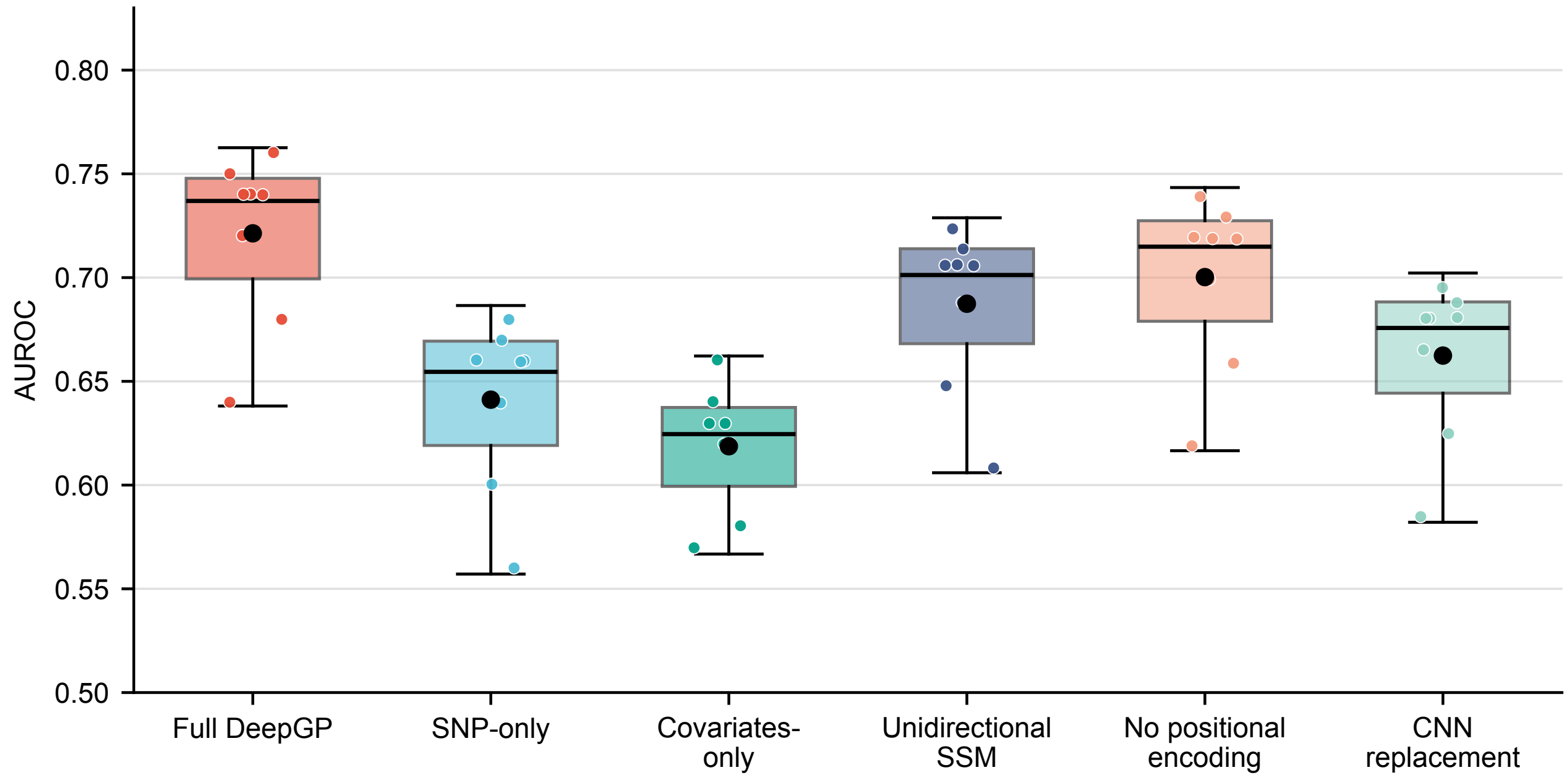

Supplementary Figure 6: Ablation analysis of DeepGP components across the eight CMDs. For each model variant, the box summarizes the distribution of AUROC over the eight cardiometabolic diseases (central line, median; box, interquartile range; whiskers, 5th–95th percentiles), the black point denotes the mean AUROC, and the coloured dots represent the individual disease-level AUROC values.

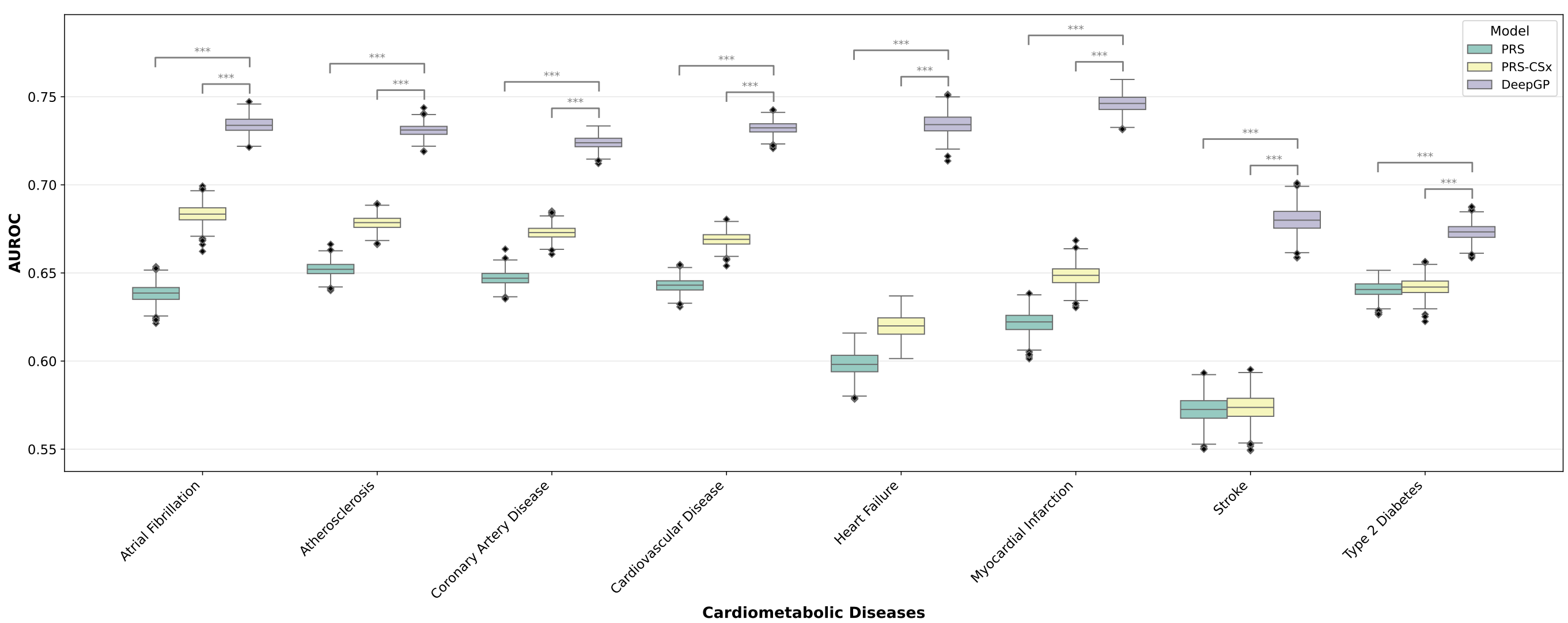

Supplementary Figure 7: AUROC under natural-prevalence evaluation. Distributions of AUROC from 1,000 stratified, paired bootstrap resamples for each model–phenotype combination. Models compared are PRS, PRS-CSx, and DeepGP across eight CMDs. Boxes denote the interquartile range (25th–75th percentiles) with the median shown as a horizontal line; whiskers span the 5th–95th percentiles. Statistical significance of DeepGP vs. each baseline was assessed with DeLong's test for correlated ROC curves; as a robustness check, paired tests on the bootstrap AUC differences reached the same conclusions. Statistical significance: $^{***}p < 0.001$, $^{**}p < 0.01$, $^{*}p < 0.05$. Sample sizes are provided in Supplementary Table 2.

can be executed on a single GPU or CPU. For machine learning approaches, we built LGBM models using LightGBM 4.3, while scikit-learn version 1.3.2 was used to implement PRS models using LR with L2 penalty, as well as RF, SNP-level LR, and MLP. To ensure a fair comparison, PRS was derived from in-house standard GWAS summary statistics generated from the training set. SNP effect sizes (β) were converted to individual scores using PLINK v2.00. We

Supplementary Table 5: AUROC comparison across DeepGP, PRS-CSx, and PRS under imbalanced (natural-prevalence) splits

Phenotype	PRS	PRS-CSx	DeepGP
Atrial Fibrillation	0.639 (0.628-0.649)	0.683 (0.674-0.694)	0.734 (0.725-0.744)
Atherosclerosis	0.652 (0.644-0.660)	0.678 (0.671-0.685)	0.731 (0.724-0.738)
Coronary Artery Disease	0.647 (0.639-0.655)	0.673 (0.665-0.680)	0.724 (0.717-0.731)
Cardiovascular Disease	0.643 (0.635-0.651)	0.669 (0.662-0.677)	0.732 (0.725-0.739)
Heart Failure	0.598 (0.585-0.612)	0.620 (0.607-0.633)	0.734 (0.723-0.744)
Myocardial Infarction	0.622 (0.611-0.634)	0.648 (0.637-0.660)	0.746 (0.736-0.756)
Stroke	0.572 (0.558-0.588)	0.574 (0.559-0.589)	0.680 (0.667-0.693)
Type 2 Diabetes	0.640 (0.631-0.650)	0.642 (0.634-0.651)	0.673 (0.665-0.682)

implemented PRS-CSx using the published implementation to obtain posterior effect sizes, which were then converted to scores. For standard PRS and PRS-CSx, the participant-level score was combined with age, sex, and the first six ancestry PCs in an LR model. DeepGP and the SNP-level baselines instead received the LD-clumped SNP dosage vector together with the same age, sex, and ancestry-PC covariates. Thus, the demographic inputs were matched across comparisons. Importantly, we applied an identical five-fold cross-validation and hyperparameter optimization protocol to all models using Optuna 3.6, ensuring methodological consistency across comparisons. For instance, we optimized key hyperparameters in LGBM, including number of estimators, learning rate, maximum tree depth, and number of leaves.

Supplementary Table 6: Ranges of hyperparameter tuning in model validation

Hyperparameter	Value
Batch size	{32, 64, 128}
Bi-SSM blocks	{1, 2, 3}
Dimension of hidden state	{16, 32, 64}
Dropout rate	[0.05 0.3]
Initial learning rate	$[1 \times 10^{-5}, 1 \times 10^{-3}]$
Number of heads in MHSA	{2,4,8}
Number of hidden layers in FCN	{1,2,4}
Weight decay	$[1 \times 10^{-5}, 1 \times 10^{-3}]$

Supplementary Table 7: Final hyperparameter configurations per CMD selected by Optuna (best AUROC)

Disease	Batch size	Bi-SSM blocks	Hidden dim	Dropout rate	Initial LR	MHSA heads	FCN layers	Weight decay
Atrial Fibrillation	128	2	32	0.12	2.5×10^{-4}	4	2	8.0×10^{-5}
Atherosclerosis	64	3	64	0.2	3.0×10^{-4}	8	2	1.0×10^{-4}
Coronary Artery Disease	64	2	64	0.18	5.0×10^{-4}	8	2	5.0×10^{-5}
Cardiovascular Disease	64	3	32	0.22	1.5×10^{-4}	4	4	2.0×10^{-4}
Heart Failure	32	2	32	0.24	7.0×10^{-4}	8	2	3.0×10^{-5}
Myocardial Infarction	128	1	32	0.10	6.0×10^{-4}	4	1	1.0×10^{-4}
Stroke	32	3	64	0.26	1.0×10^{-4}	8	4	1.5×10^{-4}
Type 2 Diabetes	64	1	32	0.2	7.0×10^{-4}	4	2	1×10^{-4}

Phenotype Definition

Based on the UKB documentation, the CMDs were defined through multiple data sources. Atherosclerosis uses hospitalization records (ICD-9 414.0, 440.*; ICD-10 I70.*, I25.0, I25.1), GP Read codes, and death registry information. Atrial fibrillation incorporates self-reported codes (field 20002), ICD-10 I48, and extensive GP Read codes. Cardiovascular disease was defined as a composite endpoint comprising three main conditions: coronary artery disease, peripheral vascular disease (ICD-9 443.8-44.9, ICD-10 I73.8-I73.9 and self-reported codes) and ischemic stroke (ICD-9

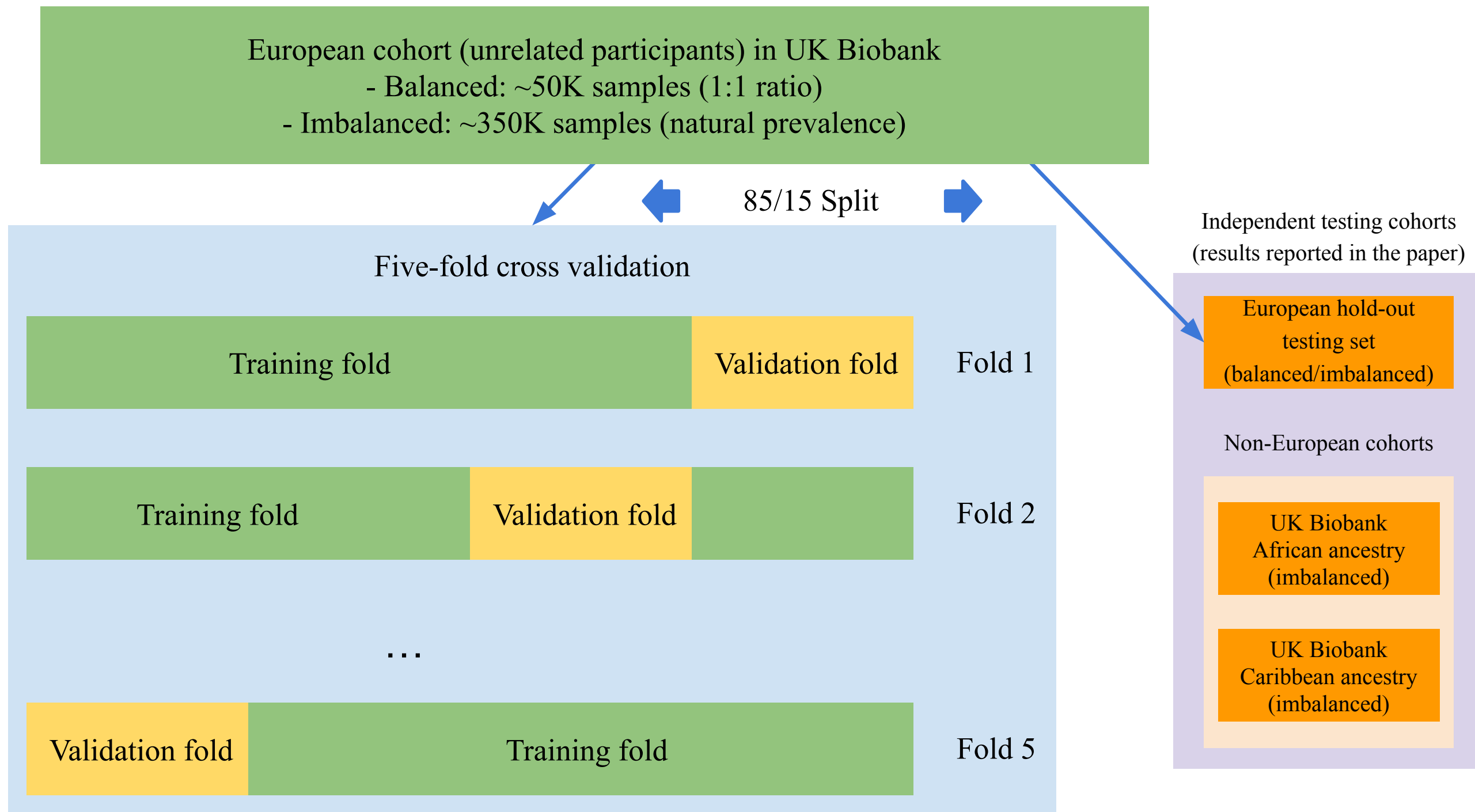

Supplementary Figure 8: Data partitioning and evaluation protocol. European-ancestry participants from UK Biobank were split 85/15 into training and testing sets. Five-fold cross-validation was performed on the training set only to select hyperparameters, while final performance is reported on fixed, independent hold-out cohorts.

433.*, 434.*, ICD-10 I63.*, and self-reported cases). Coronary Artery Disease was defined using ICD-9 410.*-412.*, 414.0, 414.8, 414.9, ICD-10 I21.*-I24.*, I25.1, I25.2, I25.5, I25.6, I25.8, I25.9, and relevant coronary intervention procedures identified through OPCS codes (K40.*-K46.*, K49, K50.1, K50.2, K50.4, K75.*). The definition was further enhanced by incorporating self-reported codes, surgical operation codes (field 20004 for PTCA/CABG/triplet-bypass procedures), and doctor-diagnosed health conditions (field 6150). Myocardial infarction uses doctor-diagnosed health conditions (heart attack), self-reported codes (heart attack), surgical operation codes, ICD-9 410.*-412.*, ICD-10 I21.*, I22.*, I24.1, I25.2, and the date of myocardial infarction (field 42000). Stroke uses ICD-9 430.*, 431.*, 434.*, 436.*, ICD-10, I60.*, I61.*, I63.*, I64.*, and self-reported codes. Type 2 diabetes was comprehensively defined through multiple data sources: self-reported diabetes (fields 20002, 2443, 4041), diabetes medication usage (fields 6153, 6177, 20003), laboratory values (HbA1c $\geq$ 6.5%, field 30750), and hospitalization records (ICD-9 code 250.* and ICD-10 codes E10.*-E14.*). Type 1 diabetes cases were specifically excluded from this definition to ensure proper phenotyping.

Glossary of Terms and Abbreviations

Table 8 presents a glossary of terms and abbreviations used in the paper, with detailed definition.

Supplementary Table 8: Glossary of key terms and abbreviations used in the paper

Term	Definition
Adam	Adaptive optimizer used for training deep models; commonly combined with weight decay and early stopping.
Accuracy	Proportion of correctly classified samples on a test set.
AUROC	Area under the receiver operating characteristic curve; a threshold-independent measure of discrimination.
Bi-SSM	Bidirectional structured state space model; scans chromosome-wise SNP sequences in forward and backward directions to model long-range intra-chromosomal dependencies.
CMD(s)	Cardiometabolic disease(s); includes atherosclerosis, atrial fibrillation, cardiovascular disease, coronary artery disease, heart failure, myocardial infarction, stroke, and type 2 diabetes.
DeepGP	Deep learning–based genome-wide predictor; a multimodal architecture that processes SNPs per chromosome with bidirectional state space modules and integrates covariates to predict cardiometabolic disease risk.
DeepLIFT	Backpropagation-based feature attribution method used to obtain SNP- and gene-level importance scores from the trained model.
FCN	Fully connected (feed-forward) network branch that processes covariates (age, sex, ancestry PCs) and serves as the final prediction head.
GWAS	Genome-wide association study; tests associations between SNPs and phenotypes, typically via linear or logistic models producing per-variant statistics.
LD	Linkage disequilibrium; non-random association of alleles at different loci that motivates LD-aware feature selection.
LD clumping	Procedure to select an index SNP per LD block and reduce correlated features (e.g., by r^2 threshold and window size).
LGBM	Light Gradient-Boosting Machine; tree-based ensemble method used as a strong machine-learning baseline.
MCC	Matthews correlation coefficient; a balanced correlation-based metric for binary classification under class imbalance.
Mamba (SSM)	A selective state space model offering Transformer-comparable capacity with linear time and memory scaling, enabling genome-scale sequence modelling.
MHSA	Multi-head self-attention; applied to chromosome-level embeddings to integrate information, including cross-chromosome dependencies, before the final prediction head.
PC(s)	Principal components of ancestry used as covariates to control for population structure.
PCA	Principal component analysis; used to visualize and adjust for ancestry differences across cohorts.
PRS	Polygenic risk score; a weighted sum of risk alleles across SNPs to estimate genetic liability to disease.
PRS-CSx	Multi-ancestry PRS method estimating posterior SNP effects under coupled continuous-shrinkage priors while modelling ancestry-specific LD.
SNP	Single-nucleotide polymorphism; a common type of genetic variant used as input features.
UKB	UK Biobank, a population-based cohort of approximately 500,000 participants from the United Kingdom with genetic and phenotypic data used for training and evaluation.
